# Differences in Salivary Flow Level, Xerostomia, and Flavor Alteration in Mexican HIV Patients Who Did or Did Not Receive Antiretroviral Therapy

**DOI:** 10.1155/2013/613278

**Published:** 2013-12-19

**Authors:** Sandra López-Verdín, Jaime Andrade-Villanueva, Ana Lourdes Zamora-Perez, Ronell Bologna-Molina, José Justino Cervantes-Cabrera, Nelly Molina-Frechero

**Affiliations:** ^1^Instituto de Investigación en Odontología, Centro Universitario de Ciencias de la Salud, Universidad de Guadalajara, 44340 Guadalajara, JAL, Mexico; ^2^Unidad de VIH del Hospital Civil de Guadalajara “Fray Antonio Alcalde”, 44340 Guadalajara, JAL, Mexico; ^3^Departamento de Investigación, Facultad de Odontología, Universidad Juárez del Estado de Durango, 34100 Durango, DGO, Mexico; ^4^Facultad de Odontología, Universidad de la República (UDELAR), 11600 Montevideo, MVD, Uruguay; ^5^Departamento de Atención a la Salud, Universidad Autónoma Metropolitana, Xochimilco, Calz del Hueso 1100 Villa Quietud, Coyoacán, 04960 Ciudad de México, DF, Mexico

## Abstract

*Introduction*. Objective and subjective alterations related to salivary flow have been reported in patients infected with human immunodeficiency virus (HIV), and these alterations are associated with the introduction of antiretroviral therapy. The aim of the current study was to discern whether these alterations are disease induced or secondary to drug therapy. *Objective*. The objective was to determine the relationships between low salivary flow, xerostomia, and flavor alterations in HIV patients who did or did not receive antiretroviral therapy. *Materials and Methods*. In this cross-sectional study, HIV patients were divided into two groups based on whether they had received antiretroviral therapy. Those patients with a previous diagnosis of any salivary gland disease were excluded. A survey was used to assess subjective variables, and colorimetry and salivary flow rates were measured using the Schirmer global test. *Results*. A total of 293 patients were included. The therapy group showed a significantly lower average salivary flow than did the group without therapy, and we observed that the flow rate tended to decrease after one year of therapy. The results were not conclusive, despite significant differences in xerostomia and flavor alteration between the groups. *Conclusion*. The study results suggest that antiretroviral therapy can cause cumulative damage that affects the amount of salivary flow.

## 1. Introduction

Oral diseases related to human immunodeficiency virus (HIV) infection have been extensively described in the clearing house classification [1] and have since been used as indicators of this condition. Additionally, both objective and subjective alterations related to salivary flow (hyposalivation, xerostomia, and dysgeusia) have been reported in these patients but have not yet been completely linked to the advent of highly active antiretroviral therapy (HAART). It is difficult to discern whether these alterations are part of the course of the disease or therapeutic side effects; various studies, which can be divided into two theories, have been performed on this subject.

On the one hand, certain authors theorize that high levels of HIV RNA might reside in the lymph nodes that are enclosed within the parotid gland during embryonic development, thus directly infecting the salivary gland with HIV [2–6]. On the other hand, others suggest an indirect process in which increased CD8+ lymphocyte infiltration into these lymph nodes could trigger significant hyperplasia in the parotid gland, which ultimately manifests as salivary gland hypofunction or enlargement of the parotid region [7–10].

Another theory about the decline in salivary flow emerged with the introduction of antiretroviral therapy in the mid-1990s. At that time, several studies reported an increase in salivary gland diseases in HIV patients, despite a decreased prevalence of oral manifestations associated with HIV and acquired immunodeficiency syndrome (AIDS) [11–17].

Clinicians should determine the reasons for decreased salivation and then make recommendations to help the patient produce more saliva and decrease those phenomena associated with saliva malfunction.

Thus, this study aimed to compare the levels of salivary flow, dry mouth sensation, and flavor alteration in newly diagnosed HIV-infected patients who did or did not receive antiretroviral therapy.

## 2. Methodology

This cross-sectional study was conducted in the HIV clinic at the Hospital Civil de Guadalajara (Mexico) “Fray Antonio Alcalde,” where an experimental group of patients receiving therapy and a control group of patients who were newly diagnosed but not receiving antiretroviral therapy were identified. Willing participants initially submitted written informed consent and later completed a survey that included inquiries about demographic data, HIV infection history, CD4+ cell counts, and viral load. Information not provided by the patients was obtained from their medical records.

**Figure 1:**
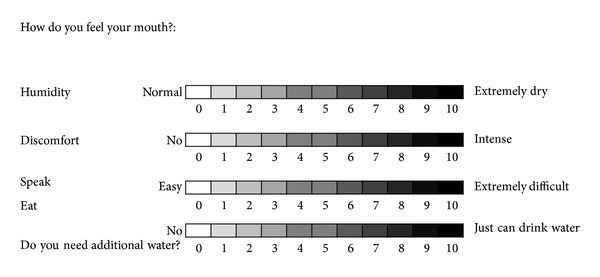
Subjective colorimetric scale for hyposalivation questions. Increased intensity is analogous to a visual scale.

The objective measurement of salivary flow levels was based on the overall Schirmer saliva test, which has set the cutoff for hyposalivation at less than 25 mm [18]. Xerostomia was assessed with a visual analog scale (from 0 to 10) that measured dryness, mouth discomfort (pain and burning in the mucosa) and difficulty speaking and eating due to a lack of saliva. A colorimetric scale (Figure 1) was used due to the low level of education in our country and associated high patient illiteracy rates. Finally, dysgeusia was assessed by the question “Have you felt changes in sweet, salty, sour or bitter taste?” If the answer was yes to any of these, the patient was considered to have an impaired sense of taste.

The control group only included patients with confirmed HIV diagnoses. The inclusion criteria for the experimental group included continuous therapy for a minimum period of 1 month. Patients who did not comply with this requirement were eliminated from the study. Patients with a previous diagnosis of salivary gland disease, neoplasia, or Sjögren syndrome were also excluded from the study.

Data were collected and analyzed with the SPSS statistical software package, version 18, and analyzed based on descriptive statistics and inferential parametric tests, including Student's *t*-test, one-way ANOVA, and the chi-squared test or Fisher's exact test (when the sample size was small), as appropriate. All statistical analyses were two tailed. A probability (*P*) value of 0.05 or less was accepted as significant.

## 3. Results

During the period from January 2011 to June 2012, 261 patients from the HIV clinic and 32 patients who were hospitalized at the Civil Hospital “Fray Antonio Alcalde” were enrolled in the study. Of these patients, 197 (67.2%) were classified into the antiretroviral therapy group, and 96 (32.8%) were classified into the control group.

According to the collected sociodemographic data, the average patient age was 37.9 ± 10.8 (range: 19–79 years). Men were more frequently represented in both study groups (*n* = 251, 85.7%) and thus, the number of women was significantly lower (*n* = 42, 14.3%). Most patients reported their marital status as single (*n* = 189, 65.9%). The group distributions of these data are shown in Table 1.

The collected information related to HIV infection showed that the most common route of infection was sexual (195, 66.6%) and that the average number of years since HIV diagnosis was 4.7 ± 9.1. Additionally, the most frequently used therapies, either alone or in combination, were nucleoside reverse transcriptase inhibitors (NRTIs).

To make comparisons between the two groups, statistical analyses related to objective and subjective evaluations were tested for the normality of salivary flow. Significant differences were observed between the averages of the groups with and without HAART. Additionally, the level of hyposalivation-related salivary flow was qualified as follows: if ≤25 mm and >25 mm, the chi-squared test was applied, which proved equal significance (Table 2), with an odds ratio of 0.557 (CI 0.323–0.962).

The patients were grouped into four categories, based on the number of years of therapy, for one-way ANOVA and intragroup assessments. The results of these analyses are shown in Table 3.

The questionnaire for subjective assessment was not administered to 26 hospitalized patients because they were unable to answer. The analysis of these data defined the values on the visual analog scale as follows: 0 = no, 1–3 = mild, 4–7 = moderate, and 8–10 = severe. By comparing the frequencies of each item (humidity, discomfort, speaking, and eating) between the two groups using the chi-squared test, a significant difference in the need to consume more fluids when eating was determined (Table 4).

Strikingly, in the subjective evaluation, patients with antiretroviral therapy mostly reported no flavor alteration, which was significantly different (*P* = 0.001) that in the group without antiretroviral therapy (Table 5).

## 4. Discussion

High concentrations of the HIV p24 antigen in the salivary gland and a viral load of greater than 10,000 copies were found to correlate with a higher prevalence of xerostomia [19–23]. These findings indicate the possibility that an increased viral load could negatively affect salivary flow. This effect was not demonstrated in this study because salivary flow was greater in the group without antiretroviral therapy (43.4 ± 27.7 versus 36.3 ± 22.2); this group also had the highest average viral load (179225.1 ± 44929.8 versus 66264.75 ± 25286.4).

In 2009, Navazesh et al. [24] proposed that a low CD4+ cell count was another risk factor for the development of salivary gland hypofunction. In contrast, in our study, the group with HAART had both the highest average CD4+ cell count (389.9 ± 260.8 versus 292.3 ± 240.5) and the lowest average salivary flow. To corroborate this finding, we categorized CD4+ cell counts according to Centers for Disease Control and Prevention (CDC) criteria [25] to determine whether a correlation existed between the salivary flow and the number of CD4+ cells. These criteria consider a low count to be fewer than 200 cells/mL, a moderate count to be between 200 and 499 cells/mL, and a high count to be at least 500 cells/mL. One-way ANOVA for these parameters found no significance (*P* = 0.468). Previously, we related changes in salivary flow to the lymphocyte count or viral load; here, antiretroviral therapy was maintained as a probable risk factor for decreased salivary flow and hyposalivation, which was corroborated by the data in Table 2.

However, we were not content with this finding and hypothesized that the patients who had received antiretroviral therapy might have experienced changes in salivary flow that were dependent on the duration of therapy. Remarkably, although previous studies had not considered such an approach, our results indicated a significant decrease in salivary flow in response to the number of years of use of antiretroviral therapy. In particular, the difference between the mean salivary flow of patients who received therapy for 1–3 years and patients who received at least 11 years of therapy was approximately 20 mm (Table 3). Obviously, to confirm this finding would require a cohort study in which various factors that could equally affect salivary flow are controlled, such as snuff use, age, and the use of other drugs that cause hyposalivation; thus, these data indicate an opportunity for further research.

Until now, protease inhibitors (PIs) were thought to be the main antiretroviral therapy responsible for low salivary flow levels and other salivary gland diseases [22]. However, the mechanism by which this antiretroviral therapy can decrease salivary flow is unclear. One suggested the possibility that the drug's chemical structure alters the structure and composition of saliva and thus reduces salivary flow [22, 26]. Another possibility is that the drug changes adipose tissue deposition within the salivary gland itself [22, 27, 28].

Because these related theories indicate that PIs are primarily responsible for salivary dysfunction, the group with HAART was subdivided into those with and those without PI use. However, no statistical significance was found when comparing the mean salivary flow (*P* = 0.820) and subjective variables between the two subgroups.

Meanwhile, subjective evaluation criteria were more frequently observed in the group without HAART because a greater proportion of these patients reported experiencing a lack of saliva when eating; thus, xerostomia was common among the patients in the higher viral load group. This finding indicated an association of antiretroviral therapy-related hyposalivation and xerostomia with viral load. It is worth mentioning that xerostomia is repeatedly confused with decreased salivary flow and is measured based on hyposalivation parameters in various studies. Thus, the methodology for xerostomia determination maybe is inadequate.

A 2009 study by Cruz et al. [29] found no significant difference in taste alteration or dysgeusia. Although this study showed no significant differences in tasting nonbitter flavors between HAART and non-HAART groups, except for separate or delimited with and without taste alteration, the frequencies of taste alteration were very similar between the groups. Additionally, it is questionable whether the patients who received HAART more frequently reported that they did not experience these alterations.

Oral manifestations include both subjective and objective parameters and are essential for diagnosis and routine health care. Hyposalivation, xerostomia, and dysgeusia are alterations that are consistent with a wide variety of diseases, syndromes, and drugs and are also easily measurable clinical parameters.

Apparently, antiretroviral therapy presents the potential risk of variations in the amount of salivary flow, and most notably, this risk increases in a therapy duration-dependent manner. However, there are clear associations of salivary flow dysregulation with subjective variables. Thus, more studies are needed to generate validated indices to measure the symptoms of dry mouth and dysgeusia and to correlate these indices with the characteristics of HIV-infected patients. In the same manner and because one limitation of this study was convenience sampling during the period of one year and a half, it would be necessary to increase the sample size for the control group.

## Figures and Tables

**Table 1:** Sociodemographic information by group.

	With HAART		Without HAART	
Age	39.6 ± 10.3		34.5 ± 11.0	
Sex	*n*	%	*n*	%
Female	26	61.9%	16	38.1%
Male	171	68.1%	80	31.9%
Marital status				
Single	125	64.8%	64	68.1%
Married	49	25.4%	21	22.3%
Divorced	15	7.8%	7	7.5%
Widowed	4	2.1%	2	2.1%

Resource: Civil Hospital “Fray Antonio Alcalde” HIV Unit.

**Table 2:** Salivary flow and hyposalivation between groups.

	With HAART		Without HAART		OR and significance
Salivary flow	Mean and DS		Mean and DS		
	36.3 ± 22.2		43.4 ± 27.7		*P* = 0.031*
Hyposalivation	*n*	%	*n*	%	
With	74	75.5%	24	24.5%	*P* = 0.047^Δ^
					OR = 0.557 (CI 0.323–0.962)
Without	122	63.2%	71	36.8%	

Resource: Civil Hospital “Fray Antonio Alcalde” HIV Unit.

^Δ^ *χ* ^2^ test; confidence interval percentage of 95%.

*Student's *t*-Test.

**Table 3:** Inter- and intragroup comparisons of years of antiretroviral therapy and salivary flow.

HAART years	Mean and standard deviation	Significance
1–3	43.4 ± 23.5	0.000^*♣*^
4–6	29.9 ± 20.1	
7–10	30.8 ± 15.9	
>11	23.4 ± 15.5	
	Mean and standard deviation	Significance
1–3		
4–6	13.5 ± 4.0	0.006^*◆*^
7–10	12.6 ± 4.5	0.048^*◆*^
>11	20.0 ± 4.5	0.000^*◆*^
4–6		
1–3	−13.5 ± 4.0	0.006^*◆*^
7–10	−0.9 ± 4.7	1.000^*◆*^
>11	6.5 ± 4.7	0.673^*◆*^
7–10		
1–3	−12.6 ± 5.4	0.048^*◆*^
4–6	0.9 ± 4.7	1.000^*◆*^
>11	7.4 ± 5.1	0.634^*◆*^
>11		
1–3	−20.6 ± 4.5	0.000^*◆*^
4–6	−6.4 ± 4.7	0.673^*◆*^
7–10	−7.4 ± 5.1	0.634^*◆*^

Resource: Civil Hospital “Fray Antonio Alcalde” HIV Unit.

^*♣*^One-way ANOVA; confidence interval percentage of 95%.

^◆^Post hoc testing: Dunnett's T3.

**Table 4:** Xerostomia evaluation between groups.

	With HAART		Without HAART		Significance
	*n*	%	*n*	%	
Humidity					
No	115	70.6	48	29.4	*P* = 0.211^Δ^
Mild	21	72.4	8	27.6	
Moderate	26	56.5	20	43.5	
Grave	17	58.6	12	41.4	
Total	**179**	**67**	**88**	**33**	
Discomfort					
No	144	67	71	33	*P* = 0.705^¶^
Mild	14	73.7	5	26.3	
Moderate	14	70	6	30	
Grave	7	53.8	6	46.2	
Total	**179**	**67**	**88**	**33**	
Speaking					
No	154	70	66	30	*P* = 0.051^¶^
Mild	13	61.9	8	38.1	
Moderate	6	37.5	10	62.5	
Grave	6	60	4	40	
Total	**179**	**67**	**88**	**33**	
Eating					
No	155	72.1	60	27.9	*P* = 0.005^Δ^
Mild	9	47.4	10	52.6	
Moderate	8	44.4	10	55.6	
Grave	7	46.7	8	53.3	
Total	**179**	**67**	**88**	**33**	

Resource: Civil Hospital “Fray Antonio Alcalde” HIV Unit.

^Δ^ *χ* ^2^ test.

^¶^Fisher's exact test.

Confidence interval percentage of 95%.

**Table 5:** Frequencies of flavor alterations between groups.

	With HAART		Without HAART		Significance
	*n*	%	*n*	%	
Flavor alteration					
Yes	20	45.5	24	54.5	*P* = 0.001^Δ^
No	159	71.3	64	28.7	
Total	**179**	**67**	**88**	**33**	
Sweet					
Yes	10	41.7	14	58.3	*P* = 0.007^Δ^
No	169	69.5	74	30.5	
Total	**179**	**67**	**88**	**33**	
Salty					
Yes	6	28.6	15	71.4	*P* = 0.000^Δ^
No	173	70.3	73	29.7	
Total	**179**	**67**	**88**	**33**	
ACID					
Yes	6	35.3	11	64.7	*P* = 0.007^Δ^
No	173	69.2	77	30.8	
Total	**179**	**67**	**88**	**33**	
Bitter					
Yes	9	47.4	10	52.6	*P* = 0.076^Δ^
No	170	68.5	78	31.5	
Total	**179**	**67**	**88**	**33**	

Resource: Civil Hospital “Fray Antonio Alcalde” HIV Unit.

^Δ^ *χ* ^2^ test.
